# Race and Sex Disparities in Lower Extremity Total Joint Arthroplasty: A Retrospective Database Study

**DOI:** 10.7759/cureus.42485

**Published:** 2023-07-26

**Authors:** Neeraj Vij, Joshua Bingham, Antonia Chen, Chase Irwin, Christian Leber, Kendall Schwartz, Kenneth Schmidt

**Affiliations:** 1 Department of Orthopaedic Surgery, University of Kansas School of Medicine - Wichita, Wichita, USA; 2 Department of Orthopaedic Surgery, Mayo Clinic, Phoenix, USA; 3 Department of Orthopaedic Surgery, Brigham and Women's Hospital, Boston, USA; 4 Department of Biostatistics, University of Arizona College of Medicine - Phoenix, Phoenix, USA; 5 Department of Medicine, University of Arizona College of Medicine - Phoenix, Phoenix, USA; 6 Department of Orthopaedic Surgery, OrthoArizona, Phoenix, USA

**Keywords:** racial disparities, knee reconstruction, hip reconstruction, equitable care, hip and knee arthroplasty, adult reconstruction

## Abstract

Introduction: Total joint arthroplasty (TJA) is successful in improving health-related quality of life. However, outcomes vary in the literature due to modifiable and non-modifiable factors. Modifiable factors consist of body mass index (BMI), nutrition, and tobacco use. Non-modifiable risk factors include age, race, sex, and socioeconomic status. Prior literature has focused on racial disparities in terms of the utilization of lower extremity arthroplasty. The purpose of this study is to determine the effect of race and sex on the in-hospital complication rate, length of stay, and charges associated with primary TJA.

Methods: This retrospective cohort utilized complex survey data from the National Inpatient Sample (NIS) between 2016 and 2019. The use of the International Classification of Disease-10 Procedure Codes (ICD-10 PCS) for right hip, left hip, right knee, and left knee TJA yielded a preliminary total of 2,660,280 patients. The exclusion criteria were bilateral arthroplasty and concomitant unilateral hip and knee arthritis. Major complications were defined as acute myocardial infarction, cardiac arrest, pulmonary embolism, adult respiratory distress syndrome, stroke, shock, and septicemia. Odds ratio (OR) and beta coefficients were adjusted for age, sex, primary payer, hospital region, hospital teaching status, and year. Total charges were adjusted for inflation using the Consumer Price Index data reported by the US Bureau of Labor Statistics.

Results: A total of 2,589,510 patients met our inclusion criteria; 87.6%, 5.9%, 4.8%, 1.4%, and 0.3% of people were ‘White’, ‘Black’, ‘Hispanic’, ‘Asian, or Pacific Islander’, and ‘Native American', as defined by the National (Nationwide) Inpatient Sample (NIS) Variable ‘RACE’. Black individuals experienced a significantly greater major complication rate compared to White individuals (0.87% vs. 0.74%, OR 1.25, p-value = 0.0004). Black and Hispanic individuals experienced a significantly greater minor complication rate compared to White individuals (6.39% vs. 4.12%, odds ratio (OR) 1.61, p-value < 0.0001; 4.68% vs. 4.12%, OR 1.17, p-value < 0.0001). Black, Hispanic, Asian or Pacific Islander, and Native American individuals stayed, on average, 0.33, 0.19, 0.19, and 0.25 days longer than White individuals (2.78, 2.54, 2.55, 2.56 vs. 2.37 days, p<0.0001). None of these statistically significant differences exceeded the established minimal clinically important difference of two days. Black, Hispanic, and Asian or Pacific Islander patients were charged $5,751, $18,656, and $12,119 more than White patients ($72,122, $85,027, $78,490, and $59,297 vs. $66,371, p ≤ 0.0165). Native American patients were charged $7,074 less than White patients ($59,297 vs. $66,371, p < 0.0001).

Conclusions: Black and Hispanic TJA patients may have higher complication rates than White TJA patients. The differences in length of stay between race groups may not affect outcomes. Hispanic patients received significantly more charges than White patients, and Native American patients received significantly fewer charges than White patients after controlling for non-modifiable risk factors. Addressing the charge disparities may reduce the total national cost burden associated with TJA. The present study highlights the need for further studies on healthcare outcomes related to race and sex.

## Introduction

Total joint arthroplasty (TJA) is successful in improving quality of life [1]. Despite its success, there is some variation in patient outcomes with modifiable and non-modifiable factors. Modifiable factors that increase the risk of complications after TJA include body mass index (BMI) > 40 kg/m2, hemoglobin A1C > 8%, poor dentition, malnutrition, *Staphylococcus aureus* colonization, and tobacco use [2]. Generally, these risk factors can be addressed preoperatively. Non-modifiable factors include race, age, sex, and socioeconomic status.

It is postulated that race plays a large role in many determinants of health, including education level, economic opportunity, poor nutrition, and environmental risks [3]. Certain racial groups have a decreased likelihood of being seen by a specialist and receive less preventative care [3]. The disparities seen by various racial groups carry over into specialty care, with racially diverse groups less likely to receive mammograms, colorectal screening, and implantable cardioverters [4-6].

These racial disparities carry over into the field of adult reconstruction. Many recent articles demonstrate a difference in the utilization rates of TJA between racial groups [7, 8]. Shahid et al. performed a comprehensive review of all primary studies on the topic and demonstrated decreased utilization among African Americans as compared to Caucasian patients [9]. Since the publication of the article, newer studies have provided an update regarding the utilization [10] and comorbidity profile [7, 8] of patients undergoing lower extremity arthroplasty. However, the published data fails to divide patients into more than two ethnic groups [7, 8]. Lastly, these studies fail to subgroup by sex and thus do not allow for commentary on the intersection between race and sex.

The purpose of this study is to determine the effect of race and sex diversity on outcomes following TJA. The primary outcome of our study was the major complication rate. The secondary outcomes are the minor complication rate, length of stay (LOS), and total charges. We hypothesize that racially diverse groups will experience higher complication rates [8], longer LOS [8], and similar total charges as compared to White patients and that these discrepancies will persist after subgrouping by sex.

## Materials and methods

Database selection

This retrospective cohort study utilized the National Inpatient Sample (NIS) [11,12]. The NIS is a large database containing summary data on inpatient hospital stays within the United States. It is one of many from the Healthcare Cost and Utilization Project (HCUP), which provides the largest publicly available all-payer inpatient databases concerning many aspects of care. The data collected represents an estimate of the true national population through the utilization of summary data and the sampling weights as determined by the NIS.

Patient population

The patient population of those aged ≥ 65 who received total hip and knee arthroplasty between 2016 and 2019 was searched on May 5th, 2022. The patient population was selected based on the codes from the International Classification of Disease-10 Procedure Codes (ICD-10 PCS) (Appendix A), which yielded a preliminary total of 2,660,280 patients. Patients undergoing bilateral arthroplasty, unilateral concomitant hip and knee arthroplasty, or patients who could not be grouped into one of the five major ethnic groups as defined by the NIS were eliminated. This maintained the same surgery across the five groups studied.

**Figure 1:**
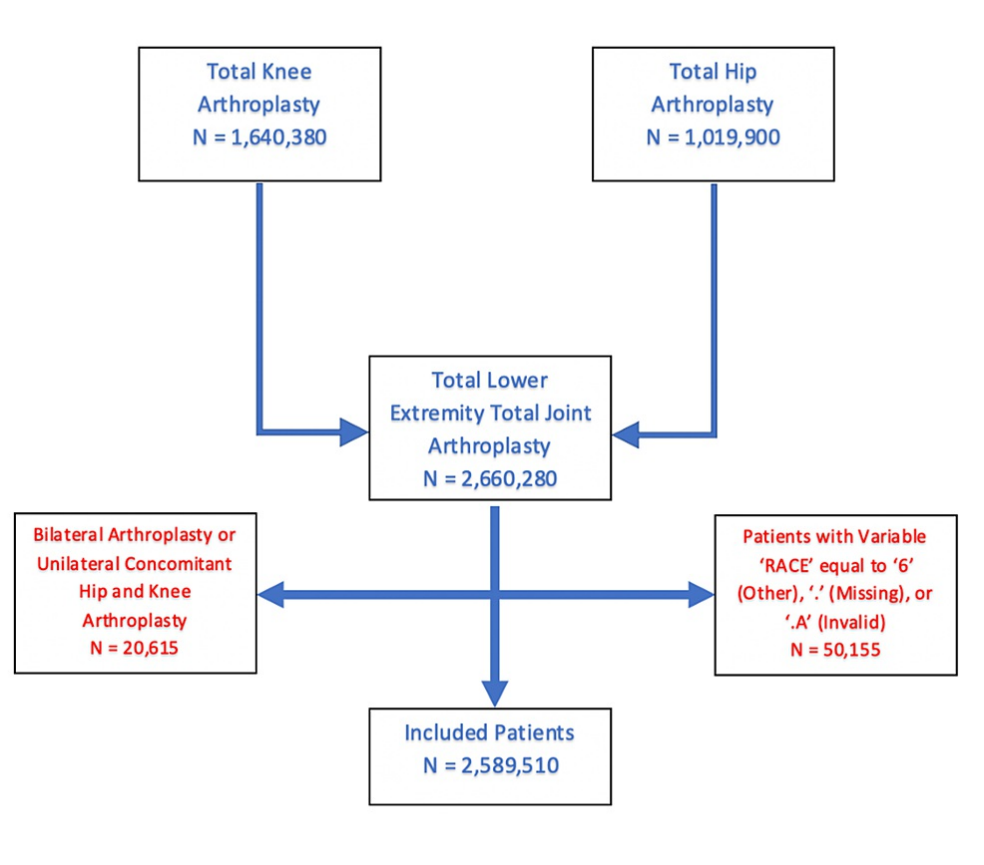
A schematic diagram depicting the application of our inclusion and exclusion criteria to the preliminary cohort to produce the final patient population.

The application of our inclusion and exclusion criteria resulted in a total of 2,589,510 patients (Figure 1).

Our study cohort was then categorized into mutually exclusive groups based upon patient race as defined by the NIS Variable ‘RACE' (‘White’, ‘Black’, ‘Hispanic’, ‘Asian or Pacific Islander’, or ‘Native American’) and ‘SEX’ ('male' or 'female') to create 10 subgroups.

Data collection

The variables collected for this study included age, race, sex, median income quartile, primary payer (Medicare, Medicaid, private insurance, self-pay, none), hospital region, hospital teaching status, LOS, total charges, and complication rate (major and minor). Charges are defined as the total dollar amount for a good or service by the involved providers and hospitals [13].

Complication rates were defined using the International Classification of Disease-10 Clinical Modification (ICD-10-CM) diagnosis codes (Appendix B) and were categorized into major and minor complications. Major complications included acute myocardial infarction, septicemia, septic shock, stroke, pulmonary embolism, adult respiratory distress syndrome (ARDS), and cardiac arrest [14]. Minor complications included urinary tract infections (UTI), pneumonia, acute renal failure, and deep venous thrombosis (DVT).

Statistical analysis

The mean and 95% confidence interval (CI) were estimated for continuous variables and the frequency and proportion for categorical variables. For the primary outcome, adjusted logistic regression models were used to estimate the odds ratio (OR) and corresponding 95% CI for experiencing a given complication. For the secondary outcomes, the adjusted linear regression models were used to estimate the beta coefficient and its respective 95% CI. Either White, White male, or White female patients were used as the reference category for the analyses, as these groups currently comprise the largest portion of the NIS database. All models were assessed for statistical assumptions and assumed independence of observations.

We assessed additional covariates for confounding and controlled for them in the primary analysis. We selected all covariates a priori based on national data and background reviews. These included patient age, primary payer information, hospital region, hospital teaching status, and year of admission. We also performed a secondary analysis to determine whether the patient LOS and total charges differed among our comparison groups. Potential confounders were controlled for, including age, sex, primary payer, hospital region, hospital teaching status, number of complications, comorbidities, and year. Both secondary outcomes were treated as continuous, and total charges were adjusted for inflation using Consumer Price Index data reported by the US Bureau of Labor Statistics. P-values were two-sided, and p < .0.05 was considered statistically significant. All p-values were independent of each other. Statistical Analysis System (SAS) version 9.4 (SAS Institute Inc., Cary, North Carolina, USA) was used for all statistical analyses.

## Results

Descriptive statistics

A total of 2,589,510 hospitalizations were associated with unilateral TJA within NIS between 2016 and 2019; there were 1,592,300 total knee arthroplasties (TKAs) and 997,210 total hip arthroplasties (THAs). The mean age was 73.14 (95% confidence interval (CI): 73.11-73.16). The White cohort consisted of 2,267,199 (87.6%) observations; 1,597,855 (61.7%) observations pertained to females. Our study cohort was evenly distributed among income quartiles, hospital regions, and the year of admission (Table 1).

**Table 1:** The demographics and patient characteristics of the 2,589,510 patients included in our study. ^a^Exclude all patients < 65 years old; ^b^Reported in US dollars, adjusted for inflation using Consumer Price Index data reported by the US Bureau of Labor Statistics; CI: confidence interval

Variables	Description (Quantitative) or Grouping (Qualitative)	Value (Mean, 95% CI or Number, Frequency)
Age^a^, Mean (95% CI)	Age of the patient in years	73.14 (73.11 – 73.16)
Race, N (%)	White	2,267,199 (87.6)
	Black	153,750 (5.9)
	Hispanic	123,225 (4.8)
	Asian or Pacific Islander	37,230 (1.4)
	Native American	8,105 (0.3)
Sex, N (%)	Male	991,655 (38.3)
	Female	1,597,855 (61.7)
Median Income Quartile, N (%)	Lowest	522,715 (20.5)
	Second	666,320 (26.1)
	Third	698,155 (27.3)
	Highest	666,215 (26.1)
Primary Payer, N (%)	Medicare	2,243,415 (86.7)
	Medicaid	13,620 (0.5)
	Private Insurance	285,755 (11.0)
	Self-Pay	7,910 (0.3)
	No Charge	335 (<0.1)
	Other	36,115 (1.4)
Hospital Region, N (%)	Northeast	491,545 (18.98)
	Midwest	638,336 (24.65)
	South	942,950 (36.41)
	West	516,679 (19.95)
Hospital Teaching Status, N (%)	Rural	239,910 (9.26)
	Urban, Non-teaching	696,635 (26.90)
	Urbann Teaching	1,652,964 (63.83)
Year, N (%)	2016	636,721 (24.59)
	2017	687,765 (26.56)
	2018	640,914 (24.75)
	2019	624,111 (24.10)
Length of Stay, Mean (95% CI)	Days stayed in the hospital	2.41 (2.39 – 2.42)
Total Charges^b^, Mean (95% CI)	Total cost of care episode	67,752 (66,902 – 68,602)

The observations associated with Medicare as their primary form of insurance were 86.7%. The average length of stay (LOS) was 2.41 days (95% CI: 2.39-2.42), and the average total charges for the hospital LOS were $67,752 (95% CI: $66,902 - $68,602).

Complications

*Racial Disparities:* *Overall Complication Rate*

The Black cohort and the Asian or Pacific Islander cohort experienced a significantly greater overall complication rate than the White cohort (6.9% vs. 4.57%, odds ratio (OR) 1.21, p-value < 0.001; 4.73% vs. 4.57%, OR 1.11, p-value < 0.0061, respectively). The individual complication rates are provided in Appendix C.

Racial Disparities: Major Complication Rate

The Black cohort experienced a significantly greater major complication rate as compared to the White cohort (0.87% vs. 0.74%, OR 1.25, p-value = 0.0004) (Table 2).

**Table 2:** The overall complication rate and grouped complication rates by ethnicity. The Caucasian group is the reference group for all odds ratios provided. The individual complication rates can be found in Appendix C. *Indicates statistically significant result for alpha = 0.05; ^a^Adjusted for age, sex, primary payer, hospital region, hospital teaching status, and year; OR: odds ratio; CI: confidence interval

Race	Number (Percentage)	Adjusted^a^ OR (95% CI)	Associated p-value
Any Complication			
Caucasian	103,670 (4.57)	Reference Group	Reference Group
African American	10,610 (6.90)	1.21 (1.16 – 1.26)	< .0001>
Hispanic/Latino	6,220 (5.05)	1.06 (0.98 – 1.13)	0.1311
Asian or Pacific Islander	1,760 (4.73)	1.11 (1.03 – 1.21)	.0061*
Native American	355 (4.38)	0.98 (0.83 – 1.16)	0.8183
Major Complications			
Caucasian	16,725 (0.74)	Reference Group	Reference Group
African American	1,330 (0.87)	1.25 (1.11 – 1.42)*	.0004*
Hispanic/Latino	865 (0.70)	0.99 (0.84 – 1.16)	0.8951
Asian or Pacific Islander	315 (0.85)	1.19 (0.93 – 1.53)	0.1642
Native American	75 (0.93)	1.39 (0.80 – 2.39)	0.2431
Minor Complications			
Caucasian	93,490 (4.12)	Reference Group	Reference Group
African American	9,830 (6.39)	1.61 (1.52 – 1.70)*	< .0001>
Hispanic/Latino	5,765 (4.68)	1.17 (1.08 – 1.26)*	< .0001>
Asian or Pacific Islander	1,560 (4.19)	1.07 (0.95 – 1.21)	0.2556
Native American	320 (3.95)	1.06 (0.82 – 1.37)	0.6692

**Figure 2:**
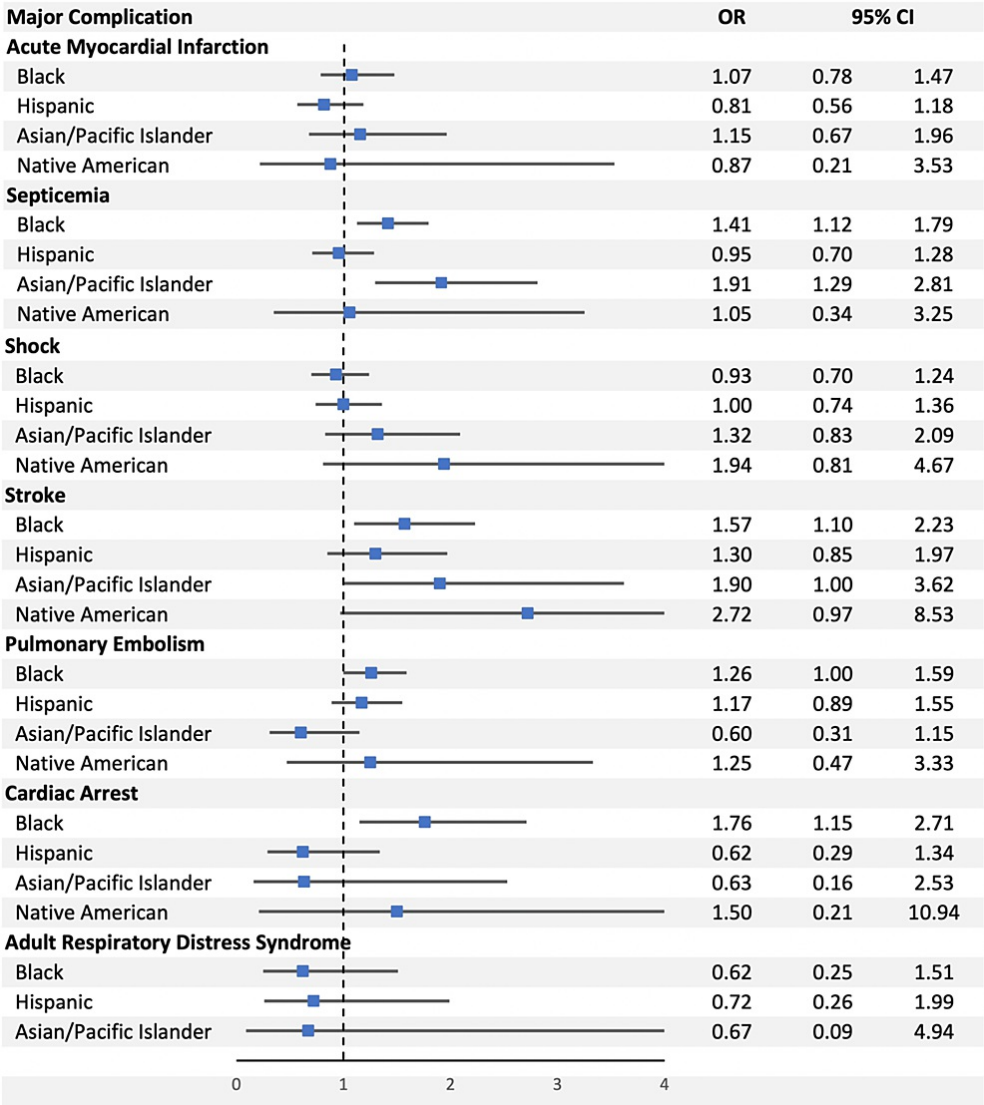
The odds ratios for the individual major complications as compared between our five major ethnic groups. OR: odds ratio; CI: confidence interval

This resulted largely from increased rates of cardiac arrest, stroke, and septicemia, whereas no differences were seen regarding rates of acute myocardial infarction, pulmonary embolism (PE), adult respiratory distress syndrome (ARDS), or shock (Figure 2).

Racial Disparities: Minor Complication Rate

**Figure 3:**
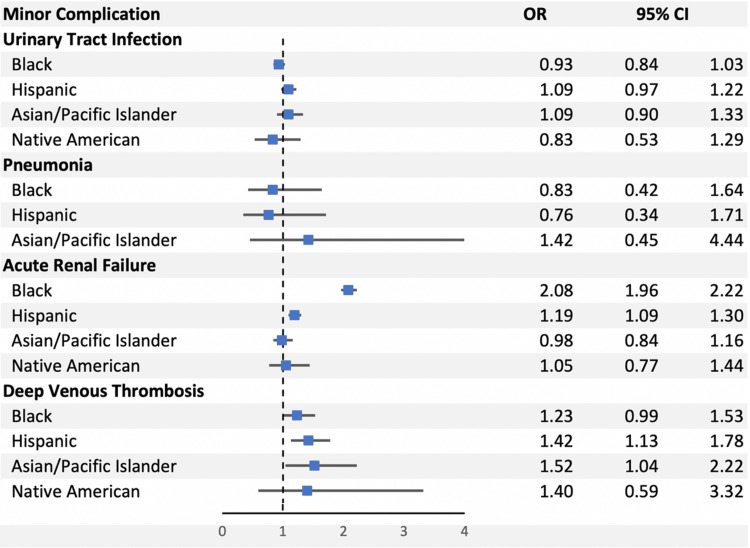
The odds ratios for the individual minor complications as compared between our five major ethnic groups. OR: odds ratio; CI: confidence interval

The Black cohort and the Hispanic cohort experienced a significantly greater minor complication rate as compared to the White cohort (6.39% vs. 4.12%, OR 1.61, p-value < 0.0001; 4.68% vs. 4.12%, OR 1.17, p-value < 0.0001) (Figure 3).

Sex Subgrouping: Overall Complication Rate

The Black male subgroup, Hispanic male subgroup, Asian or Pacific Islander male subgroup, White female subgroup, Black female subgroup, and Hispanic female subgroup experienced greater overall complication rates compared to the White male subgroup (7.39%, 5.33%, 5.06%, 4.70%, 6.69%, and 4.89% vs. 4.38%) (Table 3).

**Table 3:** The overall complication rates and the grouped complication rates by ethnicity and sex subgroups. The rate of any given complication can be seen in Appendix D. The Caucasian male group is provided as a reference group for all odds ratios provided (third column). The Caucasian female subgroup is provided as an additional reference group for the remaining female subgroups (fourth column). The individual complication rates can be found in Appendix B. *Indicates the statistically significant result for alpha = 0.05; ^a^Adjusted for age, sex, primary payer, hospital region, hospital teaching status, and year; OR: odds ratio; CI: confidence interval

Race/Sex Subgroup	N (%)	Adjusted^a^ OR Relative to White Male (95% CI)	Associated P-Value	Adjusted^a^ OR Relative to White Female (95% CI)	Associated p-value
Any Complication					
Caucasian Male	38,865 (4.38)	Reference Group	Reference Group	N/A	N/A
African American Male	3,390 (7.39)	1.80 (1.66 – 1.96)*	< .0001>	N/A	N/A
Hispanic/Latino Male	2,310 (5.33)	1.27 (1.15 – 1.41)*	< .0001>	N/A	N/A
Asian or Pacific Islander Male	565 (5.06)	1.23 (1.01 – 1.48)*	.0368*	N/A	N/A
Native American Male	140 (4.33)	1.10 (0.75 – 1.62)	0.6204	N/A	N/A
Caucasian Female	64,805 (4.70)	1.04 (1.01 – 1.08)*	.0055*	Reference Group	Reference Group
African American Female	7,220 (6.69)	1.55 (1.45 – 1.65)*	< .0001>	1.49 (1.40 – 1.58)*	< .0001>
Hispanic/Latino Female	3,910 (4.89)	1.11 (1.02 – 1.22)*	.0152*	1.07 (0.98 – 1.17)	0.1255
Asian or Pacific Islander Female	1,195 (4.59)	1.08 (0.94 – 1.24)	0.2595	1.05 (0.92 – 1.21)	0.4597
Native American Female	215 (4.41)	1.07 (0.78 – 1.47)	0.6585	1.01 (0.73 – 1.38)	0.9733
Major Complications					
Caucasian Male	7,010 (0.79)	Reference Group	Reference Group	N/A	N/A
African American Male	460 (1.00)	1.34 (1.09 – 1.67)*	.0069*	N/A	N/A
Hispanic/Latino Male	360 (0.83)	1.10 (0.86 – 1.39)	0.4474	N/A	N/A
Asian or Pacific Islander Male	120 (1.07)	1.39 (0.93 – 2.06)	0.1075	N/A	N/A
Native American Male	25 (0.77)	1.08 (0.44 – 2.61)	0.8726	N/A	N/A
Caucasian Female	9,715 (0.70)	0.86 (0.80 – 0.92)*	< .0001>	Reference Group	Reference Group
African American Female	870 (0.81)	1.04 (0.89 – 1.22)	0.6244	1.22 (1.04 – 1.42)*	.0143*
Hispanic/Latino Female	505 (0.63)	0.80 (0.64 – 0.98)*	.0318*	0.93 (0.76 – 1.15)	0.5051
Asian or Pacific Islander Female	195 (0.75)	0.94 (0.69 – 1.30)	0.7224	1.11 (0.81 – 1.52)	0.527
Native American Female	50 (1.03)	1.39 (0.74 – 2.63)	0.3052	1.61 (0.85 – 3.03)	0.1444
Minor Complications					
Caucasian Male	34,665 (3.90)	34,665 (3.90)	Reference Group	N/A	N/A
African American Male	3,125 (6.81)	3,125 (6.81)	1.85 (1.70 – 2.02)*	N/A	N/A
Hispanic/Latino Male	2,100 (4.85)	2,100 (4.85)	1.29 (1.16 – 1.44)*	N/A	N/A
Asian or Pacific Islander Male	495 (4.43)	495 (4.43)	1.20 (0.98 – 1.48)	N/A	N/A
Native American Male	130 (4.02)	130 (4.02)	1.15 (0.78 – 1.71)	N/A	N/A
Caucasian Female	58,825 (4.27)	58,825 (4.27)	1.06 (1.03 – 1.10)*	Reference Group	Reference Group
African American Female	6,705 (6.21)	6,705 (6.21)	1.60 (1.50 – 1.71)*	1.51 (1.41 – 1.61)*	< .0001>
Hispanic/Latino Female	3,665 (4.59)	3,665 (4.59)	1.17 (1.07 – 1.28)*	1.10 (1.01 – 1.20)*	.0336*
Asian or Pacific Islander Female	1,065 (4.09)	1,065 (4.09)	1.08 (0.94 – 1.25)	1.03 (0.89 – 1.20)	0.6602
Native American Female	190 (3.90)	190 (3.90)	1.06 (0.76 – 1.49)	0.98 (0.70 – 1.37)	0.8894

The individual complication rates are provided in Appendix D.

Sex Subgrouping: Major Complication Rate

**Figure 4:**
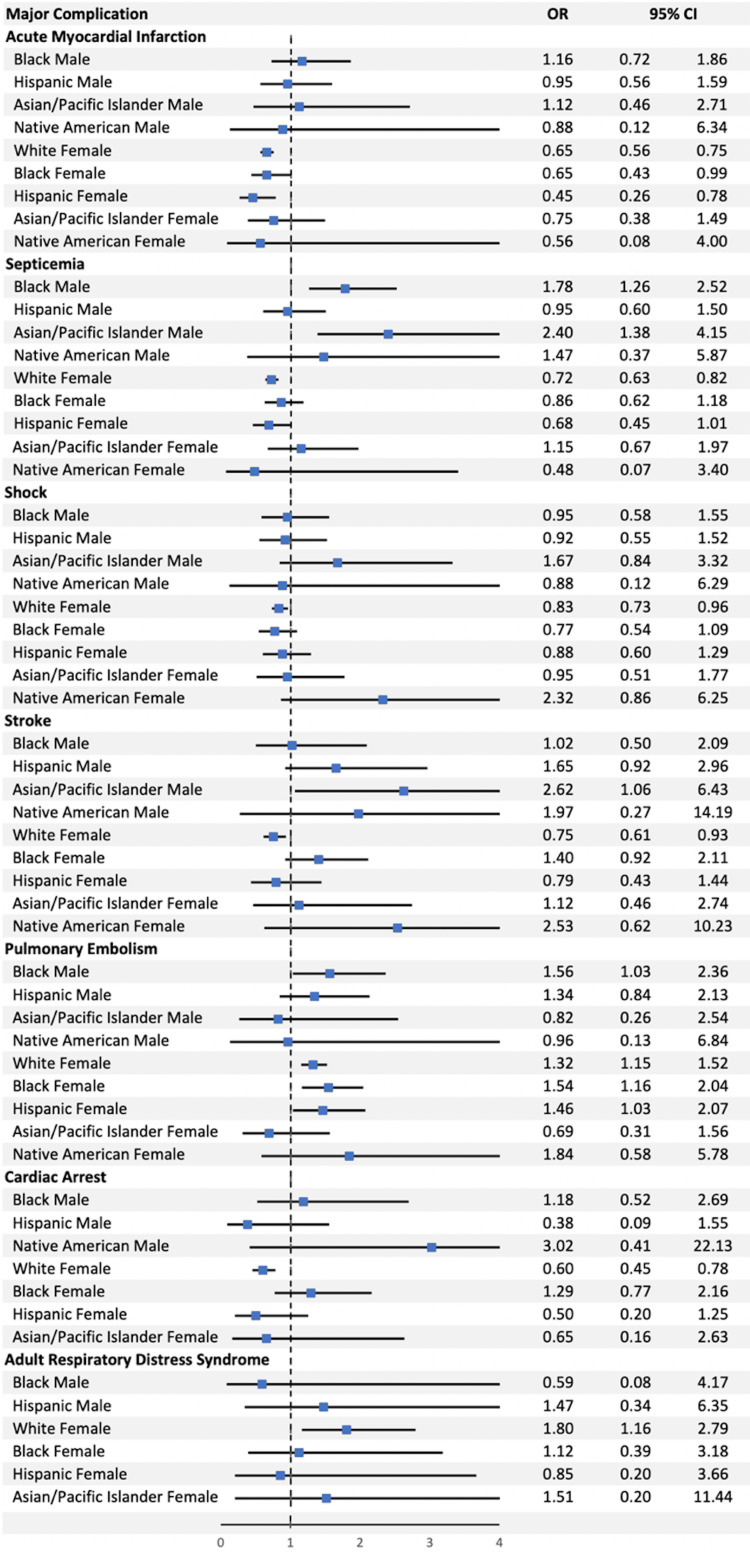
The odds ratios for individual major complications as compared between our 10 ethnic/sex subgroups. OR: odds ratio; CI: confidence interval

The Black male subgroup experienced greater major complication rates compared to the White male subgroup (1.00% vs. 0.79%, OR 1.34, p < 0.0069). The White female subgroup and Hispanic female subgroup experienced fewer major complications compared to the White male subgroup (0.70% vs. 0.79%, OR 0.86, p < 0.0001; 0.63% vs. 0.79%, OR 0.80, p-value < 0.0318) (Figure 4).

Sex Subgrouping: Minor Complication Rate

**Figure 5:**
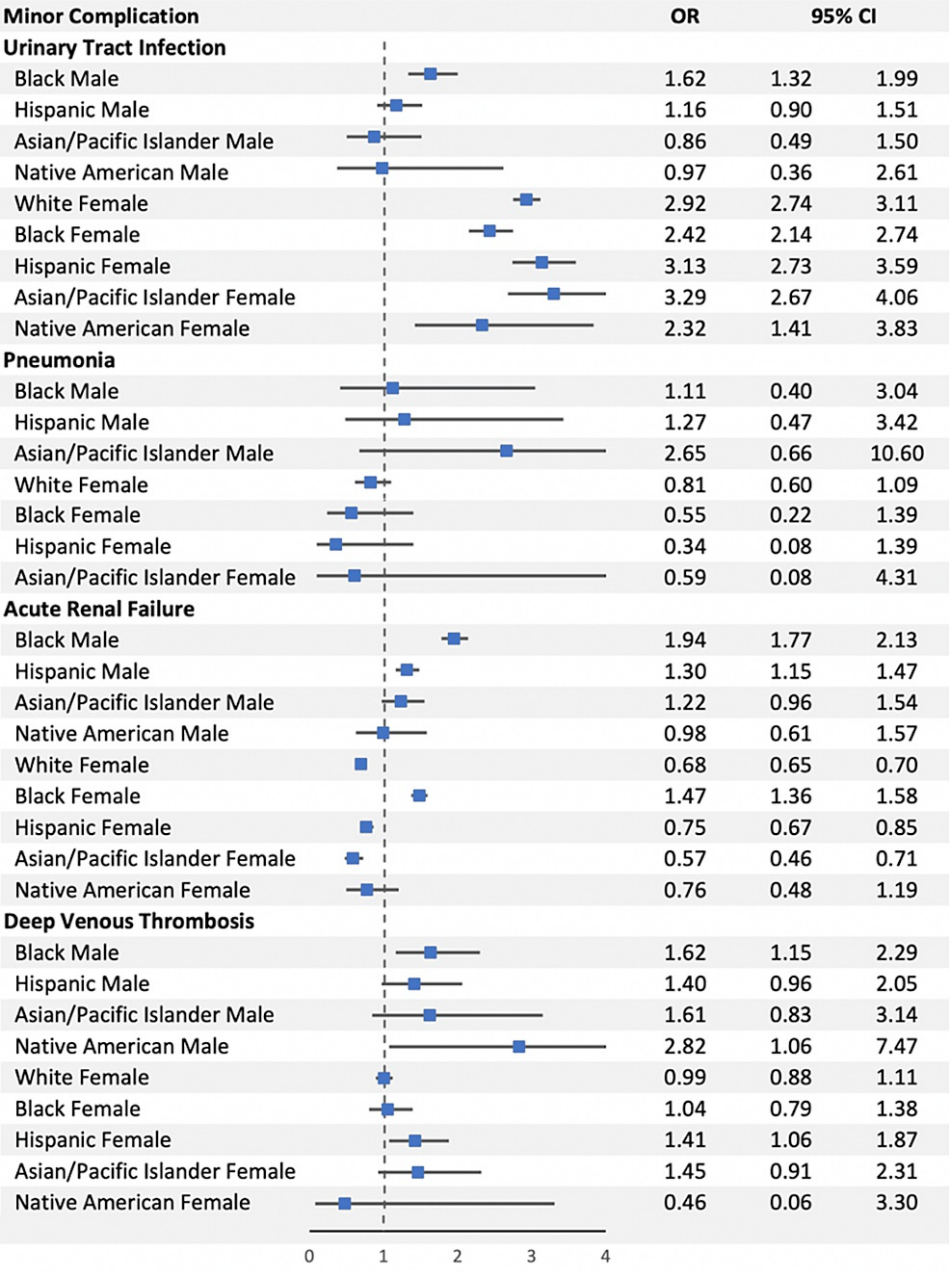
The odds ratios for individual minor complications as compared between our ten ethnic/sex subgroups.

The Black male subgroup, Hispanic male subgroup, White female subgroup, Black female subgroup, and Hispanic female subgroup experienced greater minor complication rates compared to the White male subgroup (6.81%, 4.85%, 4.27%, 6.21%, 4.59% vs. 3.90%) (Figure 5).

Length of stay

Racial Disparities

**Figure 6:**
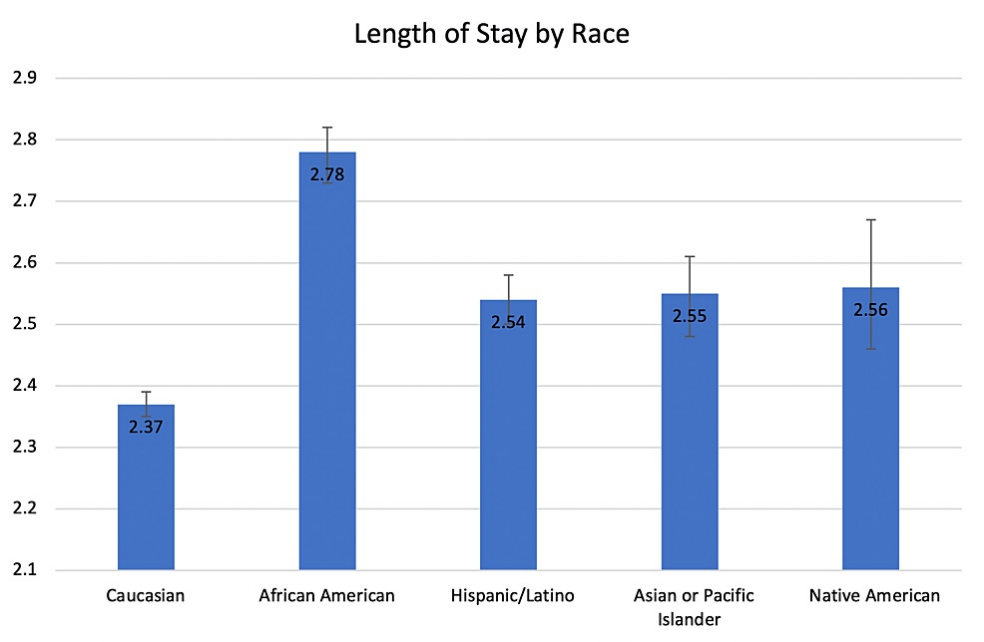
The length of stay as compared between the five major racial groups in our study.

The Black cohort, Hispanic cohort, Asian or Pacific Islander cohort, and Native American cohort stayed, on average, 0.41, 0.17, 0.18, and 0.19 days longer than the White cohort (2.78, 2.54, 2.55, 2.56 vs. 2.37 days, respectively, p < 0.0001) (Table 4, Figure 6).

**Table 4:** The length of stay as compared between the five ethnic groups in our study. The adjusted beta coefficients use Caucasians as the reference group. *Indicates statistically significant result for alpha = 0.05; ^a^Adjusted for age, sex, primary payer, hospital region, hospital teaching status, number of complications, and year; LOS: length of stay

Race	LOS(Days)	Adjusted^a^ Beta Coefficient	Associated p-value
Caucasian	2.37 (2.35 – 2.39)	Reference Group	Reference Group
African American	2.78 (2.73 – 2.82)	0.33 (0.30; 0.37)*	< .0001*
Hispanic/Latino	2.54 (2.50 – 2.58)	0.19 (0.15; 0.23)*	< .0001*
Asian or Pacific Islander	2.55 (2.48 – 2.61)	0.19 (0.13; 0.24)*	< .0001*
Native American	2.56 (2.46 – 2.67)	0.25 (0.14; 0.35)*	< .0001*

Sex Subgrouping

**Figure 7:**
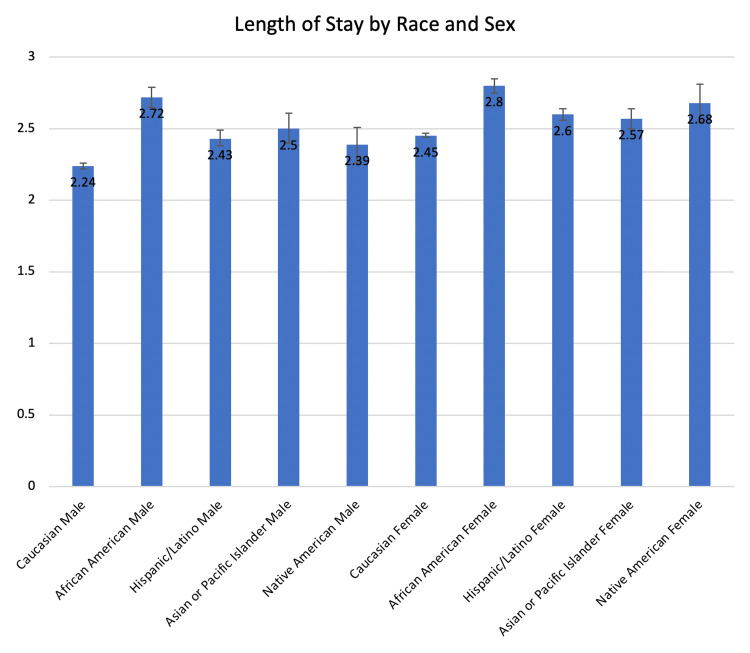
The length of stay as compared between the 10 race/sex subgroups of our study.

The Black male subgroup, Hispanic male subgroup, Asian or Pacific Islander male subgroup, and Native American male subgroup stayed 0.48, 0.19, 0.26, and 0.15 days longer than the White male subgroup (2.72, 2.43, 2.50, 2.39 vs. 2.24 days, respectively, p ≤ 0.004) (Table 5, Figure 7).

**Table 5:** The length of stay as compared between our 10 ethnic/sex subgroups. The adjusted beta coefficients are relative to the Caucasian male subgroup (third column). For all ethnically diverse female groups, the beta coefficients are additionally provided relative to the Caucasian female group (fourth column). *Indicates statistically significant result for alpha = 0.05; ^a^Adjusted for age, sex, primary payer, hospital region, hospital teaching status, number of complications, and year; LOS: length of stay

Race/Sex Subgroup	Length of Stay (Days)	Adjusted^a^ Beta Coefficient relative to Caucasian Males	Associated p-value	Adjusted^a^ Beta Coefficient relative to Caucasian Females	Associated p-value
Caucasian Male	2.24 (2.22 – 2.26)	Reference Group	Reference Group	N/A	N/A
African American Male	2.72 (2.65 – 2.79)	0.41 (0.35 – 0.46)*	< .0001*	N/A	N/A
Hispanic/Latino Male	2.43 (2.38 – 2.49)	0.23 (0.18 – 0.28)*	< .0001*	N/A	N/A
Asian or Pacific Islander Male	2.50 (2.40 – 2.61)	0.26 (0.17 – 0.36)*	< .0001*	N/A	N/A
Native American Male	2.39 (2.26 – 2.51)	0.19 (0.06 – 0.32)*	.0035*	N/A	N/A
Caucasian Female	2.45 (2.44 – 2.47)	0.17 (0.16 – 0.18)*	< .0001*	Reference Group	Reference Group
African American Female	2.80 (2.75 – 2.85)	0.47 (0.43 – 0.51)*	< .0001*	0.31 (0.27 – 0.34)*	< .0001*
Hispanic/Latino Female	2.60 (2.56 – 2.64)	0.34 (0.30 – 0.38)*	< .0001*	0.17 (0.13 – 0.21)*	< .0001*
Asian or Pacific Islander Female	2.57 (2.50 – 2.64)	0.32 (0.26 – 0.38)*	< .0001*	0.15 (0.08 – 0.21)*	< .0001*
Native American Female	2.68 (2.55 – 2.81)	0.45 (0.33 – 0.58)*	< .0001*	0.28 (0.16 – 0.41)*	< .0001*

The White female subgroup, Black female subgroup, Hispanic female subgroup, Asian or Pacific Islander female subgroup, and Native American female subgroup stayed 0.21, 0.56, 0.36, 0.33, and 0.44 days longer than the White male subgroup (2.45, 2.80, 2.60, 2.57, 2.68 vs. 2.24 days, respectively, p < 0.0001). The Black female subgroup, Hispanic female subgroup, Asian or Pacific Islander female subgroup, and Native American female subgroup stayed 0.35, 0.15, 0.12, and 0.23 days longer than the White female subgroup (2.80, 2.60, 2.57, 2.68 vs. 2.45 days, respectively, p < 0.0001).

Total charges

Racial Disparities

The Black cohort, Hispanic cohort, and Asian or Pacific Islander cohort were charged $5,751, $18,656, and $12,119 more than the White cohort ($72,122, $85,027, $78,490, $59,297 vs. $66,371, respectively, p < 0.0165) (Table 6).

**Table 6:** The total charges as compared between our five ethnic groups. The beta coefficients are provided relative to the Caucasian group. *Indicates statistically significant result for alpha = 0.05; ^a^Reported in US dollars, adjusted for inflation using Consumer Price Index data reported by the US Bureau of Labor Statistics; ^b^Adjusted for age, sex, primary payer, hospital region, hospital teaching status, number of complications, year, and length of stay

Race	Total Charges^a^ (Dollars)	Adjusted^b^ Beta Coefficient	Associated p-value
Caucasian	66,371 (65,529 - 67,213)	Reference Group	Reference Group
African American	72,122 (70,694 - 73,550)	1,876 (757; 2,995)*	.0010*
Hispanic/Latino	85,027 (83,060 - 86,995)	10,975 (9,168; 12,783)*	< .0001*
Asian or Pacific Islander	78,490 (75,868 - 81,112)	2,971 (543; 5,399)*	.0165*
Native American	59,297 (56,550 - 62,044)	-11,469 (-13,927; -9,011)*	< .0001*

**Figure 8:**
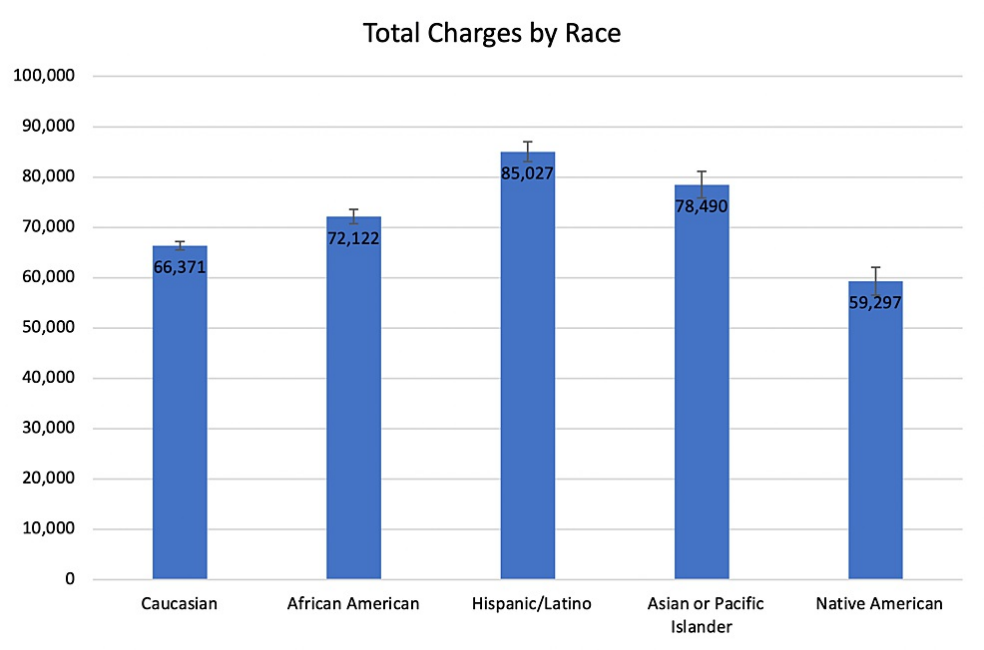
The total charges as compared between the five major racial groups of our study.

The Native American cohort was charged $7,074 less than the White cohort ($59,297 vs. $66,371, p < 0.0001) (Figure 8).

Sex Subgrouping

The Black male subgroup, Hispanic male subgroup, and Asian or Pacific Islander male subgroup were charged $6,778, $19,004, and $11,031 more than the White male subgroup ($73,526, $85,752, and $77,779 vs. $66,748, respectively, p ≤ 0.01) (Table 7).

**Table 7:** The total charges as compared between our ethnic/sex subgroups. The adjusted beta coefficients are relative to the Caucasian male subgroup (third column). For all ethnically diverse female groups, the beta coefficients are additionally provided relative to the Caucasian female group (fourth column). *Indicates statistically significant result for alpha = 0.05; ^a^Reported in US dollars, adjusted for inflation using Consumer Price Index data reported by the US Bureau of Labor Statistics; ^b^Adjusted for age, sex, primary payer, hospital region, hospital teaching status, number of complications, year, and length of stay

Race/Sex Subgroup	Total Charges^a^ (Dollars)	Adjusted^b^ Beta Coefficient relative to Caucasian Males	Associated P-Value	Adjusted^b^ Beta Coefficient relative to Caucasian Females	Associated p-value
Caucasian Male	66,748 (65,894 – 67,603)	Reference Group	Reference Group	N/A	N/A
African American Male	73,526 (71,897 – 75,155)	1,730 (355; 3,105)*	.0137*	N/A	N/A
Hispanic/Latino Male	85,752 (83,718 – 87,787)	10,742 (8,828; 12,656)*	< .0001*	N/A	N/A
Asian or Pacific Islander Male	77,779 (74,745 – 80,813)	1,403 (-1,687; 4,495)*	0.3734	N/A	N/A
Native American Male	58,863 (55,696 – 62,029)	-13,281 (-16,604; -9957)*	< .0001*	N/A	N/A
Caucasian Female	66,128 (65,283 – 66,973)	-2,409 (-2,691; -2,128)*	< .0001*	Reference Group	Reference Group
African American Female	71,525 (70,069 – 72,981)	-461 (-1,701; 778)	0.4658	2,226 (1,023; 3,429)*	.0003*
Hispanic/Latino Female	84,634 (82,653 – 86,616)	8,698 (6,775; 10,622)*	< .0001*	11,228 (9,329; 13,127)*	< .0001*
Asian or Pacific Islander Female	78,795 (76,098 – 81,491)	1,245 (-1,207; 3,696)*	0.3195	3,673 (1,247; 6,099)*	.0030*
Native American Female	59,584 (56,816 – 62,352)	-12,681 (-15,288; -10,075)*	< .0001*	-10,148 (-12,747; -7,549)*	< .0001*

**Figure 9:**
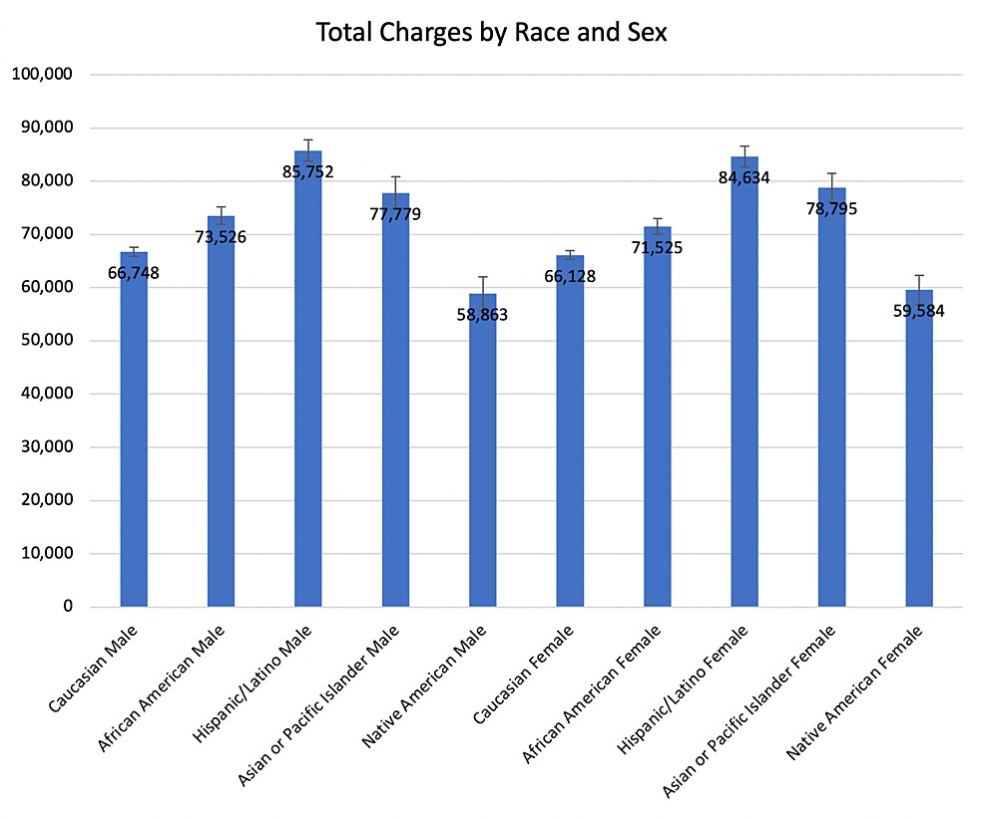
The total cost as compared between the 10 race/sex subgroups of our study.

The Native American male subgroup was charged $7,885 less than the White male subgroup ($58,863 vs. $66,748, p < 0.0001, Figure 9).

The Black female subgroup, Hispanic female subgroup, and Asian or Pacific Islander female subgroup were charged $5,397, $18,506, and $12,667 more than the White female subgroup ($71,525, $84,634, $78,795 vs. $66,128; respectively, p ≤ 0.003). The Native American female subgroup paid $6,544 less than the White female subgroup ($59,584 vs. $66,128, p < 0.0001).

## Discussion

This was a retrospective cohort study that utilized the NIS to study the population ≥ 65 years of age undergoing unilateral lower extremity TJA between 2016 and 2019. We hypothesized that racially diverse groups would experience higher complication rates, longer lengths of stay, and equivalent charges compared to the White cohort. We found that racially diverse cohorts experienced higher complication rates with persistence after subgrouping by sex. We also found that racially diverse cohorts experienced longer lengths of stay and higher total charges, which also persisted after subgrouping by sex. The exception was the Native American cohort, which experienced decreased total charges as compared to the White cohort.

In this study, we found many significant differences in complication rates among racially diverse groups compared to the White cohort. Specifically, the Black cohort was found to have significantly higher rates of cardiac arrest, stroke, septicemia, and acute renal failure. Additionally, the Hispanic cohort was found to have significantly higher rates of acute renal failure and deep venous thrombosis, while the Asian or Pacific Islander cohort experienced significantly greater deep venous thrombosis. These results are consistent with a systematic review of 82 articles relating to outcomes based on race in total hip and knee arthroplasty performed by Alvarez et al. [15]. They demonstrated that even after adjusting for comorbidities, Black individuals had higher postoperative complications compared to White individuals. It is important to note that of the 82 articles included in this study, only four examined complication rates for Asian Americans and Hispanic/Latino individuals, and none examined complication rates for Native American individuals. Similarly, they reported that Hispanic patients had a higher rate of major complications and readmission compared to White patients. However, their study found that Asian patients had similar or better outcomes in terms of major complications compared to White individuals, which is inconsistent with our finding of greater overall complication rates (contributed primarily from deep venous thrombosis) in Asian or Pacific Islander individuals. Much can be done to address these disparities, including physician education on implicit bias and the incentivization of value-based care.

When subgrouping by sex, our study generally demonstrated the persistence of racial disparities regarding complication rates, length of stay, and total charges. However, an interesting additional finding was the difference between the White male subgroup and the White female subgroup. The White female subgroup demonstrated a decreased rate of acute myocardial infarction, cardiac arrest, stroke, shock, septicemia, and acute renal failure and an increased rate of PE, ARDS, and UTI. These findings are in concordance with those of Patel et al. [16].

Subgrouping also revealed an interesting example of effect modification. The Hispanic female subgroup demonstrated an odds ratio of 1.46 concerning PE risk as compared to the White male subgroup, though no difference was seen in this complication rate between either of the race groups initially. These findings are in concordance with those of Cheah et al. [17]. This retrospective cohort of the NIS between 2006 and 2011 found an OR > 1 for women for nearly all complications studied. These findings may apply to the effect modification seen in our study; however, it is also important to consider the effect that access to care may have had.

When analyzing LOS, the data showed marginally longer lengths of stay for racially diverse groups, with persistence upon subgrouping by sex. The reasons for this are multifactorial and may include a baseline higher level of disease, access to care, and institutional-level biases. These findings are in concordance with Alvarez et al. [15] and Amen et al. [8]. However, a LOS of greater than two days has been associated with a poorer clinical outcome after lower extremity arthroplasty [18]. Thus, the statistically significant differences seen may have limited clinical significance.

The largest disparity in total charges associated with all hospital care was seen in the Hispanic cohort, who were charged $18,656 more than the White cohort after controlling for comorbidities, primary payer, and hospital region. Our study also revealed that the Native American cohort was charged $7,074 less than White individuals. Disparities in the costs of osteoarthritis (OA) treatment contribute to the total national cost burden [19] and could reduce the overall national cost by $2.3 billion [19]. The results of our study demonstrate that addressing the disparities between Hispanic and Black individuals may have the most profound effects. This may include increasing awareness of racial disparities in arthroplasty care and training on comorbidity management in diverse populations. Though our model controlled for hospital region and hospital teaching status (rural, urban: non-teaching, urban: teaching), the effect of the Urban Indian Health Program affiliation on our data remains unclear. Hospitals with this affiliation may experience differences in healthcare utilization [20] and access [21]. Subsidization of costs may also explain the difference in total charges seen in our study [22]. Further research is required to determine where the additional charges experienced by Hispanics are being incurred.

Limitations

As a retrospective cohort study, our study is unable to draw inferences regarding causality between a certain racial or race/sex subgroup and a given outcome. The complex stratification or clustered design of the NIS dataset introduces a selection bias. During the search of our patients, 50,155 patients had a race variable corresponding to 'Other', 'Missing', or 'Valid'. It is unclear whether these patients may have represented racially diverse groups and thus introduced an additional selection bias. Lastly, our study only represents complications and costs associated with the inpatient stay.

## Conclusions

Black and Hispanic TJA cohorts may have higher complication rates than the White TJA cohort. The differences in length of stay between race groups may not affect outcomes. The Hispanic cohort was charged significantly more than the White cohort, and the Native American cohort was charged significantly less than White patients. Addressing the charge disparities may reduce the total national cost burden associated with TJA. The present study highlights the need for further studies on healthcare outcomes related to race and sex.

## Appendices

Appendix A 

**Table 8:** A listing of the International Classification of Disease-10 Procedure Codes (ICD-10 PCS) used to define the study population. R: right; L: left

Clinical Entity	ICD-10 Procedure Codes Utilized
R Hip Replacement	0SR901, 0SR9019, 0SR901A, 0SR901Z, 0SR902, 0SR9029, 0SR902A, 0SR902Z, 0SR903, 0SR9039, 0SR903A, 0SR903Z, 0SR904, 0SR9049, 0SR904A,0SR904Z, 0SR906, 0SR9069, 0SR906A, 0SR906Z, 0SR90J, 0SR90J9, 0SR90JA, 0SR90JZ
L Hip Replacement	0SRB01, 0SRB019, 0SRB01A, 0SRB01Z, 0SRB02, 0SRB029, 0SRB02A, 0SRB02Z, 0SRB03, 0SRB039, 0SRB03A, 0SRB03Z, 0SRB04, 0SRB049, 0SRB04A, 0SRB04Z, 0SRB06, 0SRB069, 0SRB06A, 0SRB06Z, 0SRB0J, 0SRB0J9, 0SRB0JA, 0SRB0JZ
R Knee Replacement	0SRC06, 0SRC069, 0SRC06A, 0SRC06Z, 0SRC0J, 0SRC0J9, 0SRC0JA, 0SRC0JZ, 0SRC0L, 0SRC0L9, 0SRC0LA, 0SRC0LZ, 0SRC0M, 0SRC0M9, 0SRC0MA, 0SRC0MZ, 0SRC0N, 0SRC0N9, 0SRC0NA, 0SRC0NZ
L Knee Replacement	0SRD06, 0SRD069, 0SRD06A, 0SRD06Z, 0SRD0J, 0SRD0J9, 0SRD0JA, 0SRD0JZ, 0SRD0L, 0SRD0L9, 0SRD0LA, 0SRD0LZ, 0SRD0M, 0SRD0M9, 0SRD0MA, 0SRD0MZ, 0SRD0N, 0SRD0N9, 0SRD0NA, 0SRD0NZ

 Appendix B

**Table 9:** A listing of the International Classification of Disease-10 Clinical Modification (ICD-10-CM) codes utilized to quantify the complications of interest. 'Acute Life-Threatening' categories and ‘Other’ categories were adapted from Hustedt et al.'s 2017 definitions of ‘Major’ and ‘Minor’ complications.

Complication Category	Complication	ICD-10-CM Code
Major	Acute Myocardial Infarction	I21
	Septicemia	A40, A41
	Shock	T81.12, R65.20, R65.21, R57.0, R57.8, R57.9
	Stroke	I63, G8836, G8837, G46.4, G46.3
	Pulmonary Embolism	I26
	Adult Respiratory Distress Syndrome	J80, R06.03
	Cardiac Arrest	I46
Minor	Urinary Tract Infection	N39.0
	Pneumonia	J13, J14, J15, J16, J17, J18
	Acute Renal Failure	N17, N99.0
	Deep Venous Thromboses	I82.2, I83.3 I82.4, I82.6, I82.8, I82.9, I82.A, I82.B I82.C

Appendix C 

**Table 10:** The rate of any given complication among our study population undergoing unilateral lower extremity arthroplasty by ethnicity. The Caucasian group is the reference group for all odds ratios provided. This appendix corresponds to Table 2. *Indicates statistically significant result for alpha = 0.05; ^a^Adjusted for age, sex, primary payer, hospital region, hospital teaching status, and year

Race	Number (Percentage)	Adjusted^a^ OR (95% CI)	Associated p-value
Acute Myocardial Infarction			
Caucasian	3,680 (0.16)	Reference Group	Reference Group
African American	225 (0.15)	1.07 (0.78 – 1.47)	0.6914
Hispanic/Latino	145 (0.12)	0.81 (0.56 – 1.18)	0.2714
Asian or Pacific Islander	65 (0.17)	1.15 (0.67 – 1.96)	0.6215
Native American	10 (0.12)	0.87 (0.21 – 3.53)	0.8454
Cardiac Arrest			
Caucasian	1,055 (0.05)	Reference Group	Reference Group
African American	115 (0.07)	1.76 (1.15 – 2.71)*	.0099*
Hispanic/Latino	35 (0.03)	0.62 (0.29 – 1.34)	0.2261
Asian or Pacific Islander	10 (0.03)	0.63 (0.16 – 2.53)	0.5174
Native American	5 (0.06)	1.50 (0.21 – 10.94)	0.6874
Pulmonary Embolism (PE)			
Caucasian	4,745 (0.21)	Reference Group	Reference Group
African American	405 (0.26)	1.26 (1.00 – 1.59)	0.0502
Hispanic/Latino	290 (0.24)	1.17 (0.89 – 1.55)	0.2675
Asian or Pacific Islander	45 (0.12)	0.60 (0.31 – 1.15)	0.1247
Native American	20 (0.25)	1.25 (0.47 – 3.33)	0.6584
Acute Respiratory Distress Syndrome (ARDS)			
Caucasian	550 (0.02)	Reference Group	Reference Group
African American	25 (0.02)	0.62 (0.25 – 1.51)	0.2876
Hispanic/Latino	20 (0.02)	0.72 (0.26 – 1.99)	0.5212
Asian or Pacific Islander	5 (0.01)	0.67 (0.09 – 4.94)	0.6936
Native American	0 (0.0)	NA	NA
Stroke			
Caucasian	1,825 (0.08)	Reference Group	Reference Group
African American	175 (0.11)	1.57 (1.10 – 2.23)*	.0126*
Hispanic/Latino	115 (0.09)	1.30 (0.85 – 1.97)	0.2254
Asian or Pacific Islander	50 (0.13)	1.90 (1.00 – 3.62)	0.0508
Native American	15 (0.19)	2.72 (0.97 – 8.53)	0.0858
Shock			
Caucasian	4,205 (0.19)	Reference Group	Reference Group
African American	255 (0.17)	0.93 (0.70 – 1.24)	0.6351
Hispanic/Latino	235 (0.19)	1.00 (0.74 – 1.36)	0.9773
Asian or Pacific Islander	95 (0.26)	1.32 (0.83 – 2.09)	0.237
Native American	25 (0.31)	1.94 (0.81 – 4.67)	0.1378
Septicemia			
Caucasian	4,370 (0.19)	Reference Group	Reference Group
African American	385 (0.25)	1.41 (1.12 – 1.79)*	.0041*
Hispanic/Latino	230 (0.19)	0.95 (0.70 – 1.28)	0.7205
Asian or Pacific Islander	140 (0.38)	1.91 (1.29 – 2.81)*	0.0012
Native American	15 (0.19)	1.05 (0.34 – 3.25)	0.9367
Urinary Tract Infection			
Caucasian	33,385 (1.45)	Reference Group	Reference Group
African American	2,225 (1.45)	0.93 (0.84 – 1.03)	0.1527
Hispanic/Latino	2,015 (1.64)	1.09 (0.97 – 1.22)	0.1587
Asian or Pacific Islander	595 (1.60)	1.09 (0.90 – 1.33)	0.3749
Native American	95 (1.17)	0.83 (0.53 – 1.29)	0.4041
Pneumonia			
Caucasian	915 (0.04)	Reference Group	Reference Group
African American	45 (0.03)	0.83 (0.42 – 1.64)	0.584
Hispanic/Latino	30 (0.02)	0.76 (0.34 – 1.71)	0.5033
Asian or Pacific Islander	15 (0.04)	1.42 (0.45 – 4.44)	0.5474
Native American	0 (0.0)	NA	NA
Acute Renal Failure			
Caucasian	58,155 (2.57)	Reference Group	Reference Group
African American	7,630 (4.96)	2.08 (1.96 – 2.22)*	< .0001>
Hispanic/Latino	3,575 (2.90)	1.19 (1.09 – 1.30)*	.0002*
Asian or Pacific Islander	860 (2.31)	0.98 (0.84 – 1.16)	0.8416
Native American	190 (2.34)	1.05 (0.77 – 1.44)	0.7605
Deep Venous Thrombosis (DVT)			
Caucasian	5,540 (0.24)	Reference Group	Reference Group
African American	460 (0.30)	1.23 (0.99 – 1.53)	0.0666
Hispanic/Latino	420 (0.34)	1.42 (1.13 – 1.78)*	.0024*
Asian or Pacific Islander	140 (0.38)	1.52 (1.04 – 2.22)*	.0324*
Native American	25 (0.31)	1.40 (0.59 – 3.32)	0.4433

Appendix D 

**Table 11:** The individual complication rates among our study population undergoing unilateral lower extremity arthroplasty by ethnicity and sex subgroups. The Caucasian male group is provided as a reference group for all odds ratios provided (third column). The Caucasian female subgroup is provided as an additional reference group for the remaining female subgroups (fourth column). This appendix corresponds to Table 3. *Indicates statistically significant result for alpha = 0.05; ^a^Adjusted for age, sex, primary payer, hospital region, hospital teaching status, and year

Race/Sex Subgroup	N (%)	Adjusted^a^ OR Relative to White Male (95% CI)	Associated P-Value	Adjusted^a^ OR Relative to White Female (95% CI)	Associated p-value
Acute Myocardial Infarction					
Caucasian Male	1,785 (0.20)	Reference Group	Reference Group	N/A	N/A
African American Male	95 (0.21)	1.16 (0.72 – 1.86)	0.5541	N/A	N/A
Hispanic/Latino Male	75 (0.17)	0.95 (0.56 – 1.59)	0.8342	N/A	N/A
Asian or Pacific Islander Male	25 (0.22)	1.12 (0.46 – 2.71)	0.8058	N/A	N/A
Native American Male	5 (0.15)	0.88 (0.12 – 6.34)	0.8965	N/A	N/A
Caucasian Female	1,895 (0.14)	0.65 (0.56 – 0.75)*	< .0001>	Reference Group	Reference Group
African American Female	130 (0.12)	0.65 (0.43 – 0.99)*	.0439*	1.05 (0.70 – 1.59)	0.8117
Hispanic/Latino Female	70 (0.09)	0.45 (0.26 – 0.78)*	.0041*	0.74 (0.43 – 1.27)	0.2727
Asian or Pacific Islander Female	40 (0.15)	0.75 (0.38 – 1.49)	0.4096	1.24 (0.63 – 2.41)	0.5353
Native American Female	5 (0.10)	0.56 (0.08 – 4.00)	0.5615	0.90 (0.13 – 6.42)	0.9124
Cardiac Arrest					
Caucasian Male	530 (0.06)	Reference Group	Reference Group	N/A	N/A
African American Male	30 (0.07)	1.18 (0.52 – 2.69)	0.689	N/A	N/A
Hispanic/Latino Male	10 (0.02)	0.38 (0.09 – 1.55)	0.1788	N/A	N/A
Asian or Pacific Islander Male	0 (0.00)	NA	NA	N/A	N/A
Native American Male	5 (0.15)	3.02 (0.41 – 22.13)	0.2774	N/A	N/A
Caucasian Female	525 (0.04)	0.60 (0.45 – 0.78)*	.0002*	Reference Group	Reference Group
African American Female	85 (0.08)	1.29 (0.77 – 2.16)	0.3331	2.20 (1.32 – 3.67)*	.0027*
Hispanic/Latino Female	25 (0.03)	0.50 (0.20 – 1.25)	0.1364	0.85 (0.34 – 2.12)	0.7337
Asian or Pacific Islander Female	10 (0.04)	0.65 (0.16 – 2.63)	0.5441	1.10 (0.28 – 4.39)	0.8909
Native American Female	0 (0.00)	NA	NA	NA	NA
Pulmonary Embolism (PE)					
Caucasian Male	1,540 (0.17)	Reference Group	Reference Group	N/A	N/A
African American Male	120 (0.26)	1.56 (1.03 – 2.36)*	.0369*	N/A	N/A
Hispanic/Latino Male	95 (0.22)	1.34 (0.84 – 2.13)	0.221	N/A	N/A
Asian or Pacific Islander Male	15 (0.13)	0.82 (0.26 – 2.54)	0.7257	N/A	N/A
Native American Male	5 (0.15)	0.96 (0.13 – 6.84)	0.9634	N/A	N/A
Caucasian Female	3,205 (0.23)	1.32 (1.15 – 1.52)*	< .0001>	Reference Group	Reference Group
African American Female	285 (0.26)	1.54 (1.16 – 2.04)*	.0032*	1.18 (0.89 – 1.56)	0.2437
Hispanic/Latino Female	195 (0.24)	1.46 (1.03 – 2.07)*	.0350*	1.12 (0.79 – 1.58)	0.5251
Asian or Pacific Islander Female	30 (0.12)	0.69 (0.31 – 1.56)	0.3747	0.53 (0.24 – 1.19)	0.1236
Native American Female	15 (0.31)	1.84 (0.58 – 5.78)	0.298	1.40 (0.44 – 4.41)	0.5677
Acute Respiratory Distress Syndrome (ARDS)					
Caucasian Male	150 (0.02)	Reference Group	Reference Group	N/A	N/A
African American Male	5 (0.01)	0.59 (0.08 – 4.17)	0.5934	N/A	N/A
Hispanic/Latino Male	10 (0.02)	1.47 (0.34 – 6.35)	0.6025	N/A	N/A
Asian or Pacific Islander Male	0 (0.00)	NA	NA	N/A	N/A
Native American Male	0 (0.00)	NA	NA	N/A	N/A
Caucasian Female	400 (0.03)	1.80 (1.16 – 2.79)*	.0084*	Reference Group	Reference Group
African American Female	20 (0.02)	1.12 (0.39 – 3.18)	0.833	0.58 (0.21 – 1.60)	0.2924
Hispanic/Latino Female	10 (0.01)	0.85 (0.20 – 3.66)	0.8275	0.46 (0.11 – 1.89)	0.2826
Asian or Pacific Islander Female	5 (0.02)	1.51 (0.20 – 11.44)	0.692	0.90 (0.12 – 6.71)	0.9147
Native American Female	0 (0.00)	NA	NA	NA	NA
Stroke					
Caucasian Male	825 (0.09)	Reference Group	Reference Group	N/A	N/A
African American Male	40 (0.09)	1.02 (0.50 – 2.09)	0.9508	N/A	N/A
Hispanic/Latino Male	60 (0.14)	1.65 (0.92 – 2.96)	0.0964	N/A	N/A
Asian or Pacific Islander Male	25 (0.22)	2.62 (1.06 – 6.43)*	.0363*	N/A	N/A
Native American Male	5 (0.15)	1.97 (0.27 – 14.19)	0.5028	N/A	N/A
Caucasian Female	1,000 (0.07)	0.75 (0.61 – 0.93)*	.0075*	Reference Group	Reference Group
African American Female	135 (0.13)	1.40 (0.92 – 2.11)	0.1132	1.87 (1.24 – 2.82)*	.0030*
Hispanic/Latino Female	55 (0.07)	0.79 (0.43 – 1.44)	0.4421	1.01 (0.55 – 1.85)	0.9723
Asian or Pacific Islander Female	25 (0.10)	1.12 (0.46 – 2.74)	0.8037	1.39 (0.56 – 3.47)	0.4768
Native American Female	10 (0.21)	2.53 (0.62 – 10.23)	0.1937	3.27 (0.80 – 13.30)	0.0986
Shock					
Caucasian Male	1,795 (0.20)	Reference Group	Reference Group	N/A	N/A
African American Male	85 (0.19)	0.95 (0.58 – 1.55)	0.843	N/A	N/A
Hispanic/Latino Male	80 (0.18)	0.92 (0.55 – 1.52)	0.7328	N/A	N/A
Asian or Pacific Islander Male	40 (0.36)	1.67 (0.84 – 3.32)	0.1402	N/A	N/A
Native American Male	5 (0.15)	0.88 (0.12 – 6.29)	0.8952	N/A	N/A
Caucasian Female	2,410 (0.17)	0.83 (0.73 – 0.96)*	.0096*	Reference Group	Reference Group
African American Female	170 (0.16)	0.77 (0.54 – 1.09)	0.1441	0.91 (0.64 – 1.29)	0.5941
Hispanic/Latino Female	155 (0.19)	0.88 (0.60 – 1.29)	0.519	1.05 (0.72 – 1.53)	0.8038
Asian or Pacific Islander Female	55 (0.21)	0.95 (0.51 – 1.77)	0.8751	1.15 (0.61 – 2.16)	0.6684
Native American Female	20 (0.41)	2.32 (0.86 – 6.25)	0.0947	2.68 (1.00 – 7.19)	0.0507
Septicemia					
Caucasian Male	2,025 (0.23)	Reference Group	Reference Group	N/A	N/A
African American Male	175 (0.38)	1.78 (1.26 – 2.52)*	.0012*	N/A	N/A
Hispanic/Latino Male	95 (0.22)	0.95 (0.60 – 1.50)	0.8119	N/A	N/A
Asian or Pacific Islander Male	65 (0.58)	2.40 (1.38 – 4.15)*	.0019*	N/A	N/A
Native American Male	10 (0.31)	1.47 (0.37 – 5.87)	0.5827	N/A	N/A
Caucasian Female	2,345 (0.17)	0.72 (0.63 – 0.82)*	< .0001>	Reference Group	Reference Group
African American Female	210 (0.19)	0.86 (0.62 – 1.18)	0.3465	1.19 (0.86 – 1.63)	0.2939
Hispanic/Latino Female	135 (0.17)	0.68 (0.45 – 1.01)	0.0572	0.95 (0.63 – 1.42)	0.8017
Asian or Pacific Islander Female	75 (0.29)	1.15 (0.67 – 1.97)	0.6082	1.63 (0.94 - 2.81)	0.0827
Native American Female	5 (0.10)	0.48 (0.07 – 3.40)	0.4592	0.66 (0.09 – 4.71)	0.6801
Urinary Tract Infection					
Caucasian Male	5,945 (0.67)	Reference Group	Reference Group	N/A	N/A
African American Male	480 (1.05)	1.62 (1.32 – 1.99)*	< .0001>	N/A	N/A
Hispanic/Latino Male	335 (0.77)	1.16 (0.90 – 1.51)	0.2504	N/A	N/A
Asian or Pacific Islander Male	60 (0.54)	0.86 (0.49 – 1.50)	0.5923	N/A	N/A
Native American Male	20 (0.62)	0.97 (0.36 – 2.61)	0.9565	N/A	N/A
Caucasian Female	27,440 (1.99)	2.92 (2.74 – 3.11)*	< .0001>	Reference Group	Reference Group
African American Female	1,745 (1.62)	2.42 (2.14 – 2.74)*	< .0001>	0.82 (0.73 – 0.92)*	.0007*
Hispanic/Latino Female	1,680 (2.10)	3.13 (2.73 – 3.59)*	< .0001>	1.07 (0.94 – 1.21)	0.3364
Asian or Pacific Islander Female	535 (2.05)	3.29 (2.67 – 4.06)*	< .0001>	1.13 (0.92 – 1.39)	0.2398
Native American Female	75 (1.54)	2.32 (1.41 – 3.83)*	< .0001>	0.78 (0.48 – 1.29)	0.3397
Pneumonia					
Caucasian Male	395 (0.04)	Reference Group	Reference Group	N/A	N/A
African American Male	20 (0.04)	1.11 (0.40 – 3.04)	0.8479	N/A	N/A
Hispanic/Latino Male	20 (0.05)	1.27 (0.47 – 3.42)	0.6435	N/A	N/A
Asian or Pacific Islander Male	10 (0.09)	2.65 (0.66 – 10.60)	0.1678	N/A	N/A
Native American Male	0 (0.0)	NA	NA	N/A	N/A
Caucasian Female	520 (0.04)	0.81 (0.60 – 1.09)	0.1587	Reference Group	Reference Group
African American Female	25 (0.02)	0.55 (0.22 – 1.39)	0.2051	0.69 (0.28 – 1.74)	0.4352
Hispanic/Latino Female	10 (0.01)	0.34 (0.08 – 1.39)	0.1328	0.42 (0.10 – 1.71)	0.2261
Asian or Pacific Islander Female	5 (0.02)	0.59 (0.08 – 4.31)	0.6039	0.71 (0.10 – 5.21)	0.7337
Native American Female	0 (0.0)	NA	NA	NA	NA
Acute Renal Failure					
Caucasian Male	27,685 (3.12)	Reference Group	Reference Group	N/A	N/A
African American Male	2,640 (5.76)	1.94 (1.77 – 2.13)*	< .0001>	N/A	N/A
Hispanic/Latino Male	1,665 (3.84)	1.30 (1.15 – 1.47)*	< .0001>	N/A	N/A
Asian or Pacific Islander Male	400 (3.58)	1.22 (0.96 – 1.54)	0.099	N/A	N/A
Native American Male	85 (2.63)	0.98 (0.61 – 1.57)	0.927	N/A	N/A
Caucasian Female	30,470 (2.21)	0.68 (0.65 – 0.70)*	< .0001>	Reference Group	Reference Group
African American Female	4,990 (4.63)	1.47 (1.36 – 1.58)*	< .0001>	2.17 (2.01 – 2.34)*	< .0001>
Hispanic/Latino Female	1,910 (2.39)	0.75 (0.67 – 0.85)*	< .0001>	1.12 (1.00 – 1.26)	0.0551
Asian or Pacific Islander Female	460 (1.77)	0.57 (0.46 – 0.71)*	< .0001>	0.86 (0.70 – 1.07)	0.1817
Native American Female	105 (2.15)	0.76 (0.48 – 1.19)	0.2222	1.10 (0.70 – 1.72)	0.6927
Deep Venous Thrombosis (DVT)					
Caucasian Male	2,170 (0.24)	Reference Group	Reference Group	N/A	N/A
African American Male	180 (0.39)	1.62 (1.15 – 2.29)*	.0059*	N/A	N/A
Hispanic/Latino Male	145 (0.33)	1.40 (0.96 – 2.05)	0.0806	N/A	N/A
Asian or Pacific Islander Male	45 (0.40)	1.61 (0.83 – 3.14)	0.1585	N/A	N/A
Native American Male	20 (0.62)	2.82 (1.06 – 7.47)*	.0375*	N/A	N/A
Caucasian Female	3,370 (0.24)	0.99 (0.88 – 1.11)	0.842	Reference Group	Reference Group
African American Female	280 (0.26)	1.04 (0.79 – 1.38)	0.7596	2.17 (2.01 – 2.34)*	< .0001>
Hispanic/Latino Female	275 (0.34)	1.41 (1.06 – 1.87)*	.0199*	1.12 (1.00 – 1.26)	0.0551
Asian or Pacific Islander Female	95 (0.36)	1.45 (0.91 – 2.31)	0.1167	0.86 (0.70 – 1.07)	0.1817
Native American Female	5 (0.10)	0.46 (0.06 – 3.30)	0.4403	1.10 (0.70 – 1.72)	0.6927
